# Manipulating energy migration within single lanthanide activator for switchable upconversion emissions towards bidirectional photoactivation

**DOI:** 10.1038/s41467-019-12374-4

**Published:** 2019-09-27

**Authors:** Qingsong Mei, Akshaya Bansal, Muthu Kumara Gnanasammandhan Jayakumar, Zhiming Zhang, Jing Zhang, Hua Huang, Dejie Yu, Chrishan J. A. Ramachandra, Derek J. Hausenloy, Tuck Wah Soong, Yong Zhang

**Affiliations:** 1 Department of Biomedical Engineering, Faculty of Engineering, National University of Singapore, Singapore, 117583 Singapore; 2 School of Environmental and Chemical Engineering, Shanghai University, 99 Shangda Road, 200444 Shanghai, China; 3 Department of Physiology, Yong Loo Lin School of Medicine, National University of Singapore, Singapore, 117456 Singapore; 4 Electrophysiology core, Medical Science cluster, Yong Loo Lin School of Medicine, National University of Singapore, Singapore, 117456 Singapore; 5 Cardiovascular & Metabolic Disorders Program, Duke-National University of Singapore Medical School, Singapore, Singapore; 6 National Heart Research Institute Singapore, National Heart Centre, Singapore, Singapore; 7 Yong Loo Lin School of Medicine, National University Singapore, Singapore, Singapore; 8 The Hatter Cardiovascular Institute, University College London, London, UK; 9 The National Institute of Health Research University College London Hospitals Biomedical Research Centre, Research & Development, London, UK; 10 Tecnologico de Monterrey, Centro de Biotecnologia-FEMSA, Nuevo Leon, Mexico; 11 Neurobiology/Ageing Programme, Life Sciences Institute, National University of Singapore, Singapore, 117456 Singapore

**Keywords:** Nanobiotechnology, Nanoparticles, Optical materials and structures

## Abstract

Reliance on low tissue penetrating UV or visible light limits clinical applicability of phototherapy, necessitating use of deep tissue penetrating near-infrared (NIR) to visible light transducers like upconversion nanoparticles (UCNPs). While typical UCNPs produce multiple simultaneous emissions for unidirectional control of biological processes, programmable control requires orthogonal non-overlapping light emissions. These can be obtained through doping nanocrystals with multiple activator ions. However, this requires tedious synthesis and produces complicated multi-shell nanoparticles with a lack of control over emission profiles due to activator crosstalk. Herein, we explore cross-relaxation (CR), a non-radiative recombination pathway typically perceived as deleterious, to manipulate energy migration within the same lanthanide activator ion (Er^3+^) towards orthogonal red and green emissions, simply by adjusting excitation wavelength from 980 to 808 nm. These UCNPs allow programmable activation of two synergistic light-gated ion channels VChR1 and Jaws in the same cell to manipulate membrane polarization, demonstrated here for cardiac pacing.

## Abstract (web summary)

Orthogonal light based control of biology is of interest, yet the synthesis of materials capable of this is complex. Here, the authors report on the synthesis of simpler upconversion nanoparticles which used cross-relaxation to change emission spectra from red to green light with a change in NIR wavelength.

## Introduction

Light plays vital roles in organisms for driving a variety of important biological processes^1,2^. Its high degree of spatiotemporal resolution, and ease of precisely regulating parameters such as wavelength and intensity, also makes it an ideal external stimulus for in situ chemical and biological manipulation^1–6^. Despite these advantages, its utility as a therapeutic tool is limited by the dependence of a majority of phototherapies on ultraviolet and blue light with their limited tissue penetration, strong absorption by endogenous chromophores such as flavins, hemoglobin and melanin and strong scattering compared to longer wavelengths^7,8^. Thus, there has been a push towards a more red-shifted approach using lanthanide ion doped UCNPs. These particles convert near-infrared (NIR) excitations into visible emissions, thus enabling the activation of specific light-gated physiological functions with deep tissue-penetrating NIR light irradiation^9–11^. However, traditional UCNPs give out single colored emissions or simultaneously emit multiple colored signals under 980 nm excitation, which are only suitable for mono-directional modulations of biological processes.

A key strategy when studying biological pathways underlying diseased states is to gain bidirectional control over the signaling process. To allow programmable or bidirectional regulations of multiple biological pathways, UCNPs should exhibit orthogonal emission properties with modulation of external stimuli such as excitation wavelength^12,13^. Although there have been some attempts at generating orthogonal emissive UCNPs, the particles reported thus far deploy two or more separate activator ions for generating orthogonal emissions that are typically green and blue^12,14–16^. Owing to the complex energy level structures of these ions and the propensity for crosstalk, these activator ions need to be spatially segregated into separate layers in the resulting nanocrystals. This makes the particle structure complex and their synthesis tedious.

The complicated energy level structure of lanthanide ions can be an asset and not a disadvantage, if one can precisely navigate the energy transfer pathways within single lanthanide ions to favor discrete energy transitions. This challenge once overcome would eliminate the need for using multiple lanthanide ions for obtaining orthogonal emissive properties^12,14–16^. For example, Er^3+^ ions are able to produce green color luminescence through the ^2^H_11/2_, ^4^S_3/2_ → ^4^I_15/2_ transition, and red emission from the ^4^F_9/2_ → ^4^I_15/2_ transition^14,15,17,18^. The energy transition processes for green emissions and red emissions of Er^3+^ ions simultaneously occur after the photon is pumped into excited states in the typical UCNPs.

In order to selectively realize different energy transitions under disparate external stimuli, we have developed a CR-mediated switchable upconversion luminescence. The energy migration within the same activator Er^3+^ ions is precisely manipulated to obtain orthogonal emissive UCNPs that emit the green and red emissions, simply by altering excitation wavelengths from 980 nm to 808 nm. We believe that these red and green emissions being red-shifted compared to UV or blue light, are more suited for biological applications. In addition, excitation wavelengths in the NIR range, allows modulations to be carried out at deeper tissues. Moreover, by circumventing the crosstalk that results from multiple activator ions, these single activator doped particles show precise control over the emission profile and ratios of emitted peaks, allowing for bidirectional activation to be achieved in a programmable manner. This is demonstrated through programmable activation of two synergistic red-shifted light-gated ion channels namely, Jaws and VChR1 (responding to red and green light, respectively) expressed in the same cell to manipulate the influx of intracellular chloride and calcium ions, resulting in corresponding, on-demand membrane hyperpolarization or depolarization outcomes. The clinical potential of such manipulations is evidenced when the same ion channels expressed in cardiomyocytes were used to gain bidirectional control over beating rate, when irradiated with 980 or 808 nm light, in the presence of our orthogonal emission UCNPs.

## Results

### Design and characterization of orthogonal emission UCNPs

**Fig. 1:**
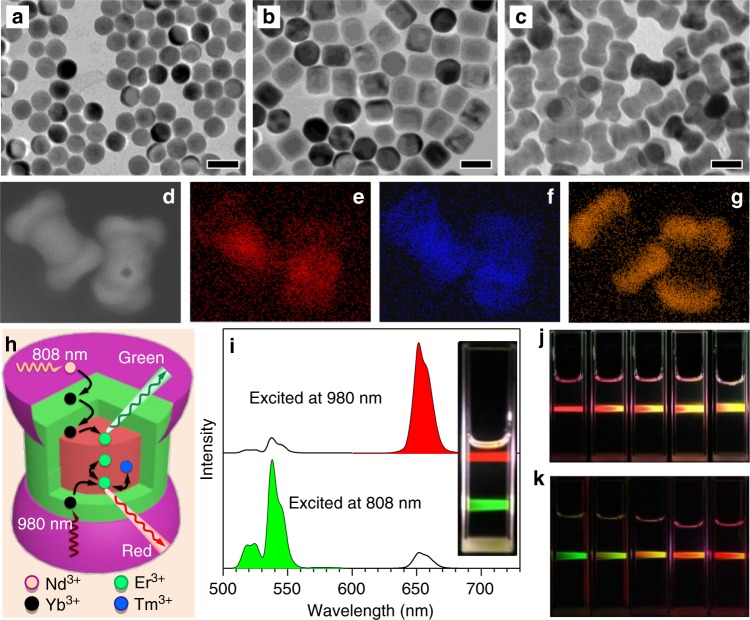
Tm^3+^ doped orthogonal emission UCNPs. **a–c** TEM images of the Tm^3+^ doped core, core@shell and the dumbbell shaped NPs. Scale bars are 50 nm. **d–g** HAADF image and corresponding elemental mapping of Er^3+^, Yb^3+^, and Nd^3+^ ions. **h** Energy migration process of the Tm^3+^ doped UCNPs when irradiated with 980 nm or 808 nm NIR lasers. **i** Upconversion luminescence spectra of the nanocrystals under excitation with a 980 nm or 808 nm continuous-wave lasers. Inset image is the corresponding photo showing red and green emission colors upon NIR excitation. **j** Photos of the Tm^3+^ doped UCNPs in cyclohexane when excited at a fixed 980 nm laser power (1 W) with increasing 808 nm laser power from 0 W to 3 W. **k** Photos of the Tm^3+^ doped UCNPs in cyclohexane when excited at a fixed 808 nm laser power (2 W), while increasing the 980 nm laser power from 0 W to 2.5 W

In our UCNP design scheme, the luminescence activator Er^3+^ was incorporated into the core structure. Yb^3+^ and Nd^3+^ ions acting as efficient sensitizers were confined in the inner shell (Yb^3+^) and outer shell (Nd^3+^) to harvest 980 nm and 808 nm excitation light, respectively. As shown in Fig. 1a and Supplementary Fig. 1, the obtained NaYbF_4_: Er^3+^/Tm^3+^ core nanoparticle was spherical; however, it became cylindrical after the inner shell NaYF_4_: Yb^3+^ coating (Fig. 1b), and finally dumbbell shaped after the outer shell NaNdF_4_: Yb^3+^ coating (Fig. 1c). X-ray diffraction patterns in Supplementary Fig. 2 demonstrated the obtained UCNPs were indexed to the hexagonal phase nanocrystals. Elemental mapping images in Fig. 1d–g indicated that the outmost shell grew longitudinally at the ends of NaYbF_4_: Er^3+^/Tm^3+^ @ NaYF_4_: Yb^3+^ nanoparticles. The formation of dumbbell shaped nanoparticles should be ascribed to the lattice mismatch between the NaNdF_4_-based outer shell and NaYF_4_-based inner shell. The lattice strain at the interface caused tremendous stress between the inner shell and outer shell, which prevented the NaNdF_4_ shell from directly growing on the NaYF_4_ inner shell, but longitudinally grew at the ends of NaErF_4_@NaYF_4_. The resulting UCNPs demonstrated orthogonal emission property, with red and green emissions arising from the same lanthanide activator, Er^3+^. Upon excitation with a 980 nm laser, Yb^3+^ ions both in the core and shell layers could absorb this excitation energy, transferring it to Er^3+^ and further to neighboring Tm^3+^ ions to give out red light. However, under excitation with 808 nm light, the vast majority of excitation energy was absorbed by Nd^3+^ ions in the outmost shell, subsequently migrating to neighboring Yb^3+^ and Er^3+^ ions to emit green light (Fig. 1h). The red emission at 650 nm was about seven-fold in intensity compared to the emission at 540 nm when excited with a 980 nm continuous-wave (C.W.) diode laser. The same UCNPs could produce green emission color in which the emission intensity at 540 nm was about seven-fold higher than that at 650 nm after harvesting 808 nm excitation light (Fig. 1i). The luminescence photo inserted in Fig. 1i further reiterated the orthogonal emission properties of these particles. In addition, the emission profile of these particles could be modulated through the programmable adjustment of 980 nm and 808 nm laser powers. As shown in Supplementary Fig. 3, the emission intensities at 540 nm increased upon increasing 808 nm laser powers from 0 W to 3 W when the 980 nm laser power was fixed at 1 W, changing the luminescence color of the UCNPs gradually from red to yellow (Fig. 1j). Likewise, the emission intensities at 650 nm also increased when increasing 980 nm laser powers from 0 W to 2.5 W, while keeping the 808 nm laser power fixed at 2 W. The resulting luminescence color changed from green to yellow, and eventually turned to red (Fig. 1k).

The doping amounts of Yb^3+^ and Er^3+^ in the core structure are crucial for modulating the orthogonal emissions. An elevated doping amount of Er^3+^ ions in the NaYF_4_ host lattice decreases interatomic distances, and thus facilitates the cross relaxation between the excited levels of ^2^F_7/2_ and ^4^I_11/2_, resulting in the decrease of green light emissions (^2^H_11/2_, ^4^S_3/2_ → ^4^I_15/2_)^19–21^. A wide range of doping concentrations of Yb^3+^ ions in the nanoparticle core, varying from 0 to 49.5%, were studied to understand the dependence of red and green emission ratios on the amounts of Yb^3+^ ions. As shown in Supplementary Fig. 4–5, the intensity ratios of red to green emissions (R/G) at 980 nm excitation decreased dramatically when Yb^3+^ doping concentration increased from 0% to 19.5%, reaching a plateau thereafter. However, the intensity ratios of green to red emissions (G/R) at 808 nm excitation exhibited a significant increase at 19.5% Yb^3+^ doping. Therefore, an ideal proportion of 19.5% Yb^3+^ with 80% Er^3+^ was established to ensure optimal orthogonal emissions. In addition to Yb^3+^ doping amounts, Tm^3+^ also plays a vital role in achieving such an orthogonal emissive property. It was found that without Tm^3+^ doping in the core, the UCNPs demonstrated green colored emission regardless of excitation with 980 nm or 808 nm lasers (Supplementary Fig. 6–8). The intensity ratios of R/G at 980 nm excitation dramatically increased with increasing Tm^3+^ doping amounts, but the intensity ratios of G/R at 808 nm excitation exhibited the opposite trend. Thus, the optimal doping amount of Tm^3+^ was chosen to be 0.5%.

### Elucidation of the energy transfer mechanism

**Fig. 2:**
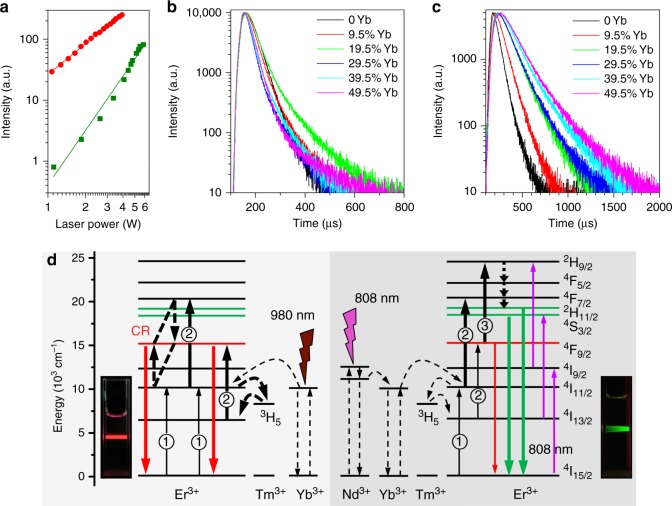
Luminescence properties of UCNPs after excitation with 980/808 nm lasers. **a** Log-log plots of the 650 nm (red line) and 540 nm (green line) luminescence intensities for the Tm^3+^ doped UCNPs under 980 nm and 808 nm excitations, respectively. **b** Luminescence decay curves of Er^3+^ ions measured at 650 nm (^4^F_9/2_ → ^4^I_15/2_) for the nanocrystals with different doping amounts of Yb^3+^ in the core upon excitation with 980 nm lasers. **c** Luminescence decay curves of Er^3+^ ions measured at 540 nm (^4^S_3/2_ → ^4^I_15/2_) for the nanocrystals with different doping amounts of Yb^3+^ in the core upon excitation with 808 nm lasers. **d** Two-photon upconversion mechanism for red emission under 980 nm laser excitation (left panel), and three-photon upconversion process for green emission under 808 nm laser excitation (right panel)

Multiphoton transition processes in UCNPs can be confirmed by linear fits of logarithm of the emission intensities vs. excitation power, where the fitted slope represents the number of excitation photons^12,22^. The red line in Fig. 2a and spectral evolutions in Supplementary Fig. 9 showed the 980 nm excitation power dependence of luminescence intensities of the UCNPs. By increasing excitation power from 1 to 4 W, the red emission increased accordingly, and the fitted linear slope for the intensities at 650 nm vs. 980 nm excitation power was 1.67, showing a two-photon transition process. The green line in Fig. 2a and spectral evolutions in Supplementary Fig. 10 demonstrated that increasing excitation power of 808 nm laser also induced linear increase in emission intensities at 540 nm, and the fitted slope was 2.96, exhibiting a three-photon transition process. To verify the luminescent mechanism, lifetime variations of the UCNPs with increasing Yb^3+^ doping amounts from 0% to 49.5% were further investigated. The results in Fig. 2b showed that the lifetimes of red emission at 650 nm exhibited a small variation upon increasing the Yb^3+^ concentration. This suggested that the energy transfer process for red emissions was not influenced by Yb^3+^ ions. However, lifetimes of the green emission at 540 nm in Fig. 2c gradually increased from 64 μs to 190 μs when the Yb^3+^ doping amounts increased from 0% to 49.5% (meanwhile, the doping amounts of Er^3+^ decreased from 99.5 to 50%), which indicated that a decrease of Er^3+^ ions prolonged the energy transfer pathway and slowed down the conversion of excitation energy into luminescence emission. Moreover, compared with the lifetimes of red emissions under 980 nm irradiation (45 μs for 19.5% Yb^3+^ dopant), the lifetimes of green emissions at 808 nm excitation (140 μs for 19.5% Yb^3+^ dopant) were much longer, indicating green emissions underwent a longer energy migration pathway.

Based on these studies, we have proposed a detailed energy transfer pathway for these Tm^3+^ doped orthogonal emission UCNPs. As shown in Fig. 2d, upon excitation with a 980 nm laser, Er^3+^ ions and Yb^3+^ ions both in the core and in the shell can absorb the excitation energy. The heavy doping of Er^3+^ (80 mol %) facilitates the occurrence of cross-relaxation between the neighboring excited Er^3+^ ions: ^4^F_7/2_ + ^4^I_11/2_ → 2 ^4^F_9/2_, resulting in red emissions. In addition, Tm^3+^ ions trap the pumped energy at the ^3^H_5_ state of Tm^3+^, subsequently back transferring it to the ^4^I_13/2_ state of Er^3+^ ions. This is followed by energy pumping with a second 980 nm photon to the ^4^F_9/2_ state of Er^3+^ ions, leading to an enhancement of red emission at 650 nm. In case of excitation with 808 nm light, given that the extinction coefficient of Nd^3+^ at 808 nm is much higher than that of Er^3+^ (Supplementary Fig. 11), the vast majority of excitation energy is absorbed by Nd^3+^ ions, which are distributed at the ends of the dumbbell shaped particles. According to the lifetime variations mentioned above, the green emissions rely on the following energy transfer process, Nd^3+^ → Yb^3+^ → Er^3+^, to transport the absorbed energy from outmost shell to inner core, which is a distance-dependent transfer process^23^. This unique feature causes the excitation energy to be preferentially transferred to the adjacent Er^3+^ ions and greatly decreases the population of neighboring excited Er^3+^ ions, attenuating the probability of CR. Although, the doped Tm^3+^ ions can trap partial excitation energy and back transfer it to Er^3+^, the pumping probability with a second 980 nm photon to the ^4^F_9/2_ state of Er^3+^ becomes much smaller under this 808 nm excitation scheme. In addition, very little excitation energy of 808 nm is directly absorbed by Er^3+^ ions, and migrates along the pathway indicated by the purple lines. Therefore, CR process and energy trapping effect of Tm^3+^ ions does not significantly affect the emissions when excited with 808 nm light, leading to mainly green light emission.

This hypothesis was further validated by the fact that in the absence of the outer Nd^3+^ containing shell, the nanoparticles exhibited red emission at 808 nm excitation (Supplementary Fig. 12). The excitation energy at 808 nm was solely absorbed by Er^3+^ ions in the core-shell structured nanoparticles (NaErF_4_: Yb^3+^/Tm^3+^ @ NaYF_4_:Yb^3+^), which greatly enhanced the probability of the CR process and made the nanoparticles exhibit red emissions. However, after coating with the outmost shell (NaNdF_4_:Yb^3+^), majority of the excitation energy was absorbed by Nd^3+^ ions, followed by migration to the neighboring Er^3+^ ions, resulting in green emissions. Moreover, if the doping amounts of Yb^3+^ ions in the first shell were increased, more Er^3+^ ions in the core would be pumped into excited states, thus enhancing the intensities of red emissions (Supplementary Fig. 13).

### Bidirectional photoactivation using orthogonal emission UCNPs

Next, we wanted to ascertain whether these nanoparticles with excitation wavelength dependent orthogonal emission could be used for on-demand photoactivation of multiple photoresponsive moieties in the same cell. As a proof of concept, we chose to apply these particles towards bidirectional control of light-gated proteins. Our motivation for seeking bidirectional control stems from the complexity of biological pathways involved in disease pathology. Being able to achieve bidirectional control over a biological process can be instrumental in not only treating a diseased state, an example being cardiac pacing for arrhythmias, but also in understanding the how and why of their occurrence. Bidirectional control over cell types such as neurons could pave the way to interrogating neuronal circuits in a precise manner, isolating the function of discrete neuronal populations. However, the poor tissue penetration of blue light makes deeper regions of the body inaccessible, resulting in a concerted effort towards development of better light targeting strategies as well as red-shifted opsins^4^. For this study, we chose two candidate light-gated ion channels, VChR1 and Jaws that are red-shifted versions of the commonly used opsins, Channelrhodopsin (ChR2) and Halorhodopsin (NpHR)^24,25^. These studies were designed to ascertain whether our orthogonal emission UCNPs could be used for precise bidirectional control over the activation of these two ion channels, by switching between the two NIR excitation wavelengths (980 nm and 808 nm). VChR1 is a cation conducting ion channel belonging to the channelrhodopsin family. Upon irradiation with green light (540–589 nm), this ion channel allows cations like Ca^2+^, H^+^, Na^+^ to flood in, resulting in membrane depolarization. In excitable cells like neurons and cardiomyocytes, this can result in firing of action potentials. This in turn can be used for reversing diseased states such as peripheral paralysis through direct stimulation of motor neurons, correcting cardiac arrhythmias etc^26,27^. Jaws is a Cl^-^ conducting ion channel belonging to the halorhodopsin family. Upon irradiation with red light (630–650 nm), this ion channel allows influx of Cl^−^ ions, resulting in membrane hyperpolarisation and neuronal inhibition^25,28–31^. Such neuronal inhibition has been used for isolating swim command centers in zebrafish, for studying epilepsy, in restoring light sensitivity to cone cells etc. Bidirectional control over both these ion channels in the same cell offers a multitude of therapeutic possibilities such as building optogenetic pacemakers, in managing neuropsychiatric disorders like anxiety and in studying the neural circuitry underlying disease states^32,33^.

UCNPs were first tested for their cytotoxicity to ensure they can be used for further biological studies. HEK293T cells were incubated with different concentrations of UCNPs ranging from 0.1 to 1.5 mg/mL and the cell viability was tested after 24 h. As shown in Supplementary Fig. 14a, cells exposed to UCNPs concentrations up to 1 mg/mL exhibited negligible toxicity whereas moderate toxicity was observed upon exposure to 1.5 mg/mL of UCNPs. So the concentration of 1 mg/mL was chosen as the optimal amount for further studies, ensuring sufficient UCNPs were present for efficient photoactivation without significant toxicity. Apart from testing UCNPs toxicity, neither of the two NIR wavelengths (980 or 808 nm) caused any significant toxicity to the cells for all the exposure durations tested (Supplementary Fig. 14b). The minimal reduction in fluorescence intensity upon continuous irradiation using both 980 and 808 nm NIR lasers indicated their excellent photostabilities (Supplementary Fig. 15). The rise in temperature with 980 and 808 nm NIR irradiation was tested on different substrates (water, cell culture media, cell culture media + cells) and there was a maximum increase of ~4 °C in the cells + media group after irradiation for 120 s as shown in Supplementary Fig. 16.

HEK293T cells were transfected with Jaws and/or VChR1 to study the photo-modulation of these ion channels. The characterization of both the plasmids is given in Supplementary Fig. 17. We studied whether the red or green emissions of these particles evoked by 980 nm or 808 nm excitation, respectively, could activate the corresponding light responsive ions channels Jaws or VChR1. This was done through fluorescence measurements and electrophysiology studies. In the fluorescence response studies, the channel activation was inferred from the change in fluorescence of ion sensitive dyes. Rhod-4 is a Ca^2+^ responsive dye that fluoresces brighter upon binding to Ca^2+^, serving as a measure of VChR1 channel activation^14^. Similarly, MQAE is a chloride sensitive dye that is quenched in response to increasing Cl^−^ concentration. Thus, by measuring decrease in MQAE fluorescence, the activation of Jaws could be inferred. Optimization studies were conducted to ascertain the amount of UCNPs needed, incubation duration and NIR laser exposure time for maximum ion channel activation (Supplementary Fig. 18–19). There was a decrease in VChR1 activation in cells incubated with UCNPs for periods longer than 60 mins before irradiation with 808 nm light. In addition, a UCNP concentration of at least 0.75 mg/ml and NIR irradiation duration of at least 60 s were needed for VChR1 activation. Based on these results, we fixed the UCNPs concentration for further studies as 1 mg/ml with an incubation time of 10–60 min and NIR light (808 or 980 nm) irradiation time of 60 s.

**Fig. 3:**
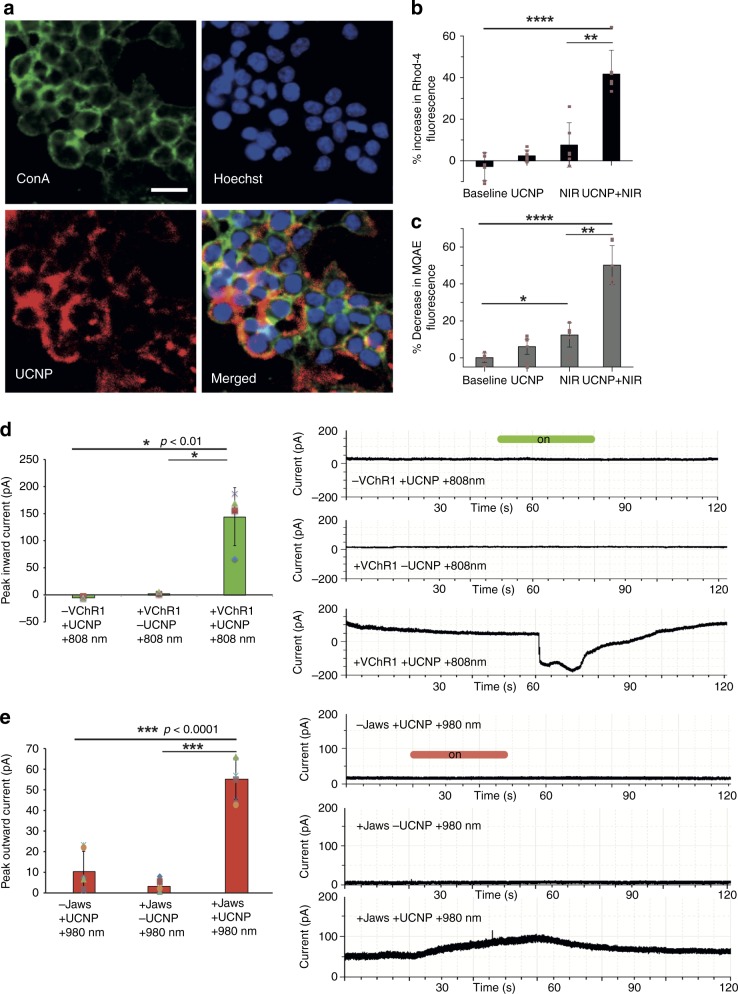
Photoactivation using orthogonal emissive UCNPs. **a** UCNPs uptake by HEK293T cells. UCNPs show red fluorescence. Cell membrane is stained green and the nucleus blue. Scale bar is 50 μm. **b**, **c** Activation of VChR1 and Jaws in HEK293T cells expressing these proteins, upon irradiation with 808 nm or 980 nm light, respectively, in the presence of UCNPs. VChR1 activation is indicated by the increase in fluorescence of the Ca^2+^ responsive dye, Rhod-4, and Jaws activation is indicated by a decrease in fluorescence of the Cl^-^ responsive dye, MQAE. **d**, **e** show whole-cell patch-clamp results of voltage clamped (0 mV) VChR1 or Jaws expressing HEK293T cells. Graphs showing peak inward currents in response to irradiation of VChR1 expressing cells (*n* = 4) with 808 nm light in the presence of UCNPs (emitting green light) and peak outward currents in Jaws expressing HEK293T cells (*n* = 6) when irradiated with 980 nm light in the presence of the same UCNPs (emitting red light). These are accompanied by current traces of representative samples. Controls include unmodified cells irradiated with NIR light (980 or 808 nm) in the presence of UCNPs or transfected cells irradiated with NIR light in the absence of UCNPs. Error bars represent the standard deviation of measurements. *p < 0.01(ANOVA), **p < 0.001(ANOVA), ***p < 0.0001(ANOVA), ****p < 0.00001(ANOVA)

Confocal images of HEK293T cells incubated with UCNPs for 60 min showed majority of UCNPs present on the cell surface (Fig. 3a). Using the parameters fixed previously, it was seen that exposing cells transfected with either VChR1 or Jaws plasmids (Supplementary Fig. 20) and incubated with UCNPs to either 808 nm or 980 nm light, resulted in successful activation of the respective ion channels, evidenced by the fluorescence changes of Rhod-4 and MQAE in the representative subtraction images (Supplementary Fig. 21). As controls, cells were untreated, incubated with UCNPs but unexposed to NIR light or exposed to NIR light in the absence of UCNPs. None of these groups showed appreciable changes in fluorescence of either Rhod-4 or MQAE dyes, indicating that the fluorescence changes in test group (UCNPs + NIR) were attributed to the activation of VChR1 (Fig. 3b) or Jaws (Fig. 3c) by the upconverted green or red light of the UCNPs when excited by 808 nm or 980 nm light, respectively. This was further confirmed by the results of our electrophysiology study. HEK293T cells transfected with VChR1 when exposed to 808 nm light (green emission) in the presence of UCNPs showed a strong inward current indicative of cation influx, which returned to baseline upon cessation of the light stimulus. Such a response was not observed in untransfected cells exposed to light in the presence of UCNPs or in transfected cells exposed to light in the absence of UCNPs (Fig. 3d). Similarly, cells transfected with the Jaws plasmid showed an outward current indicative of anion influx when exposed to 980 nm light (red emission) in the presence of UCNPs. This returned to baseline upon cessation of the light stimulus. Such a response was not observed in untransfected cells exposed to light in the presence of UCNPs or in transfected cells exposed to light in the absence of UCNPs (Fig. 3e). These results indicate that the green and red emissions evoked from our orthogonal emission UCNPs upon the 808 and 980 nm excitation can activate VChR1 and Jaws, respectively.

**Fig. 4:**
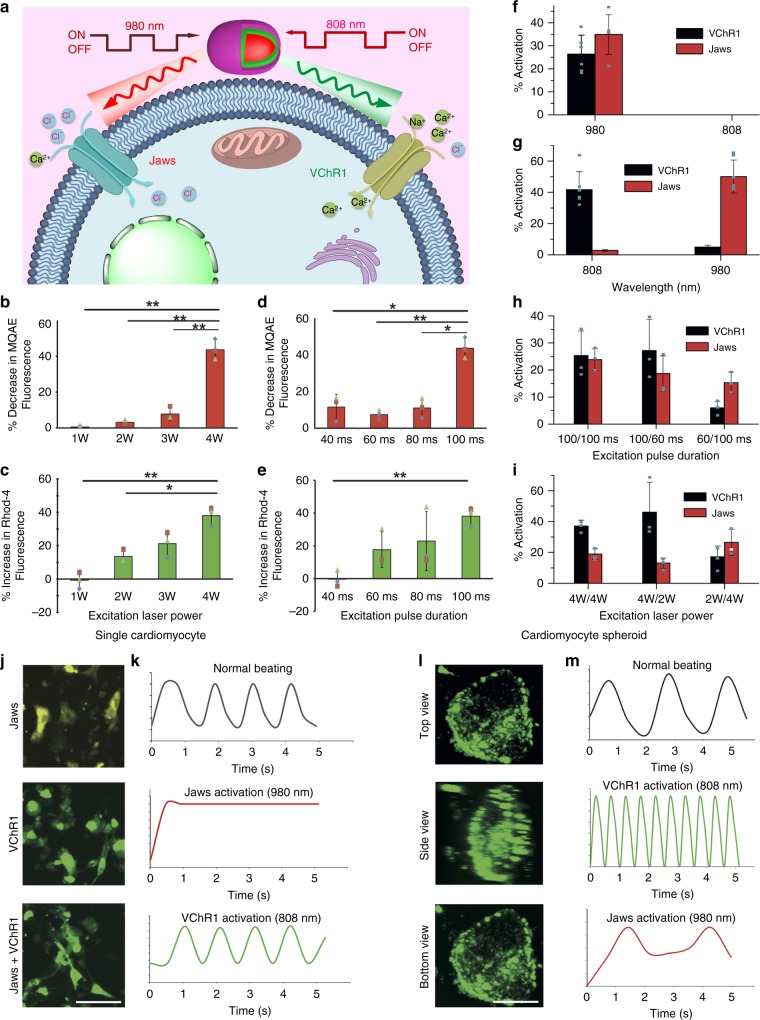
Programmable Photoactivation. **a** Schematic illustration of programmable activation of ion channel proteins Jaws and VChR1 through controlling the power and duration times of 980 nm and 808 nm lasers. The green emission produced upon 808 nm excitation of the UCNPs activates VChR1 resulting in cation (calcium ion) influx, while the red emission produced by 980 nm excitation activates Jaws for anion (chloride ion) influx. **b**, **c** Activation of Jaws (**b**) and VChR1 (**c**) expressed in HEK293T cells by varying 980 nm and 808 nm laser powers (*n* = 3). **d**, **e** Activation of Jaws (**d**) and VChR1 (**e**) expressed in HEK293T cells by varying pulse duration time of 980 nm and 808 nm lasers (*n* = 3). **f** Simultaneous activation of VChR1 and Jaws in co-transfected cells with traditional Yb^3+/^Er^3+^ UCNPs as compared to sequential activation of these proteins by the orthogonal emissive UCNPs (**g**). **h**, **i** Programmable activation of ion channel proteins Jaws and VChR1 through controlling the power and duration times of 980 nm and 808 nm lasers. **j** Expression of VChR1 and Jaws in IPSC derived cardiomyocytes as seen by the co-expression of YFP and GFP, respectively, followed by expression of both plasmids in co-transfected cells. Scale bar is 50 µm. **k** Change in beating rate of the cardiomyocytes (*n* = 3) co-transfected with Jaws and VChR1 upon irradiation with 980 and 808 nm lasers, respectively, in the presence of orthogonal emissive UCNPs. **l** Top, side and bottom view of Z-stack confocal image of cardiomyocyte spheroid co-transfected with VChR1 and Jaws. The green fluorescence is indicative of GFP and EYFP expression. Scale bar is 200 µm. **m** Change in beating rate of the cardiomyocyte spheroids (*n* = 3) co-transfected with Jaws and VChR1 upon irradiation with 980 and 808 nm lasers, respectively, in the presence of orthogonal emissive UCNPs. Error bars represent the standard deviation of measurements *p < 0.01 (ANOVA), **p < 0.001(ANOVA)

Following this, we wanted to investigate whether these UCNPs could be used for bidirectional and programmable control in the same cell. The schematic in Fig. 4a describes the proposed mechanism. The light sensitive ion channels VChR1 and Jaws are expressed in HEK293T cells. VChR1 responds to green light, resulting in an influx of cations such as Ca^2+^ and Na^+^ leading to membrane depolarization, while Jaws is a redshifted anion channel belonging to the halorhodopsin family of opsins which, results in Cl^−^ influx and membrane hyperpolarization in response to red light. When the cell expressing these ion channels is incubated with the emission switchable UCNPs, irradiation with 808 nm light causes green upconverted emission and VChR1 activation, while irradiation with 980 nm light causes red upconverted emission and activation of Jaws. The UCNPs were distributed uniformly in the cells within 60 min of incubation (Supplementary Fig. 22). Greater change in MQAE fluorescence was observed with an increase in 980 nm irradiation power and excitation pulse duration. Maximum channel activation was seen at an excitation pulse duration of 100 ms and excitation power of 4 W (corresponding to an average power density of 0.5 W/cm^2^). A similar trend was seen when cells were irradiated with 808 nm light for VChR1 activation (Fig. 4b–e).

The photoactivation seen with these orthogonal emission UCNPs is different from that seen with traditional UCNPs that emit both green and red light at 980 nm excitation. To highlight the difference, we synthesized traditional NaYF_4_:Yb^3+^/Er^3+^ UCNPs and used them for VChR1 and Jaws activation. With these particles both channels were activated upon irradiation with 980 nm light (since the particles emit both red and green light at this wavelength), while no activation was seen with 808 nm excitation (Fig. 4f). Thus, with these particles, sequential, on-demand, temporally controlled activation of the two channels cannot be achieved, with both channels getting activated simultaneously. However, with the orthogonal emission UCNPs, varying the NIR excitation wavelength changed the ion channel being activated. Upon irradiation with light at 808 nm, the VChR1 ion channel was activated with no significant Jaws activation, while with 980 nm excitation, Jaws was activated with no significant VChR1 activation (Fig. 4g). Thus sequential, temporally controlled activation of two distinct proteins co-expressed in the same cell using the same particles could be achieved. In addition, programmable activation of the two ion channels could be attained in cells expressing both, by varying the power densities and pulse durations of the respective NIR excitation lights. Keeping the excitation powers of both 808 nm and 980 nm lasers constant at 4 W, and simply changing the excitation pulse duration time resulted in variable activation levels of these two ion channels (Fig. 4h). Similarly, keeping the excitation pulse duration constant and varying the excitation powers of the two NIR light lasers also resulted in variable activation of the two ion channels (Fig. 4i). The above results indicate that these UCNPs allow sequential, on demand and programmable activation of multiple ion channels in the same cell type, a property that is very advantageous for manipulation of biological processes where bidirectional and programmable control of cells is needed.

To illustrate the therapeutic potential of such particles, we used them for controlling the beating rate of induced pluoripotent stem-cell (IPSC)-derived cardiomyocytes. These cardiomyocytes, either plated as monolayers or as clusters of cells (spheroids), were co-transfected with Jaws and VChR1, using lipofectamine 3000. The transfection protocol was optimized and the cells showed good expression levels of both ion channels (Fig. 4j). In the cardiomyocyte clusters, to ensure that these proteins were expressed throughout the spheroid and not just on the surface, Z-stack confocal imaging was done. The top, side and bottom views of the rendered Z-stack image depicted in Fig. 4l show plasmid expression (Jaws + VChR1) throughout the spheroid, as indicated by the green fluorescence of the fused GFP/EYFP proteins. A PDMS disc incorporated with UCNPs (as shown in Supplementary Fig. 23) was used for photoactivation of 2D cultures and 3D spheroids of cardiomyocytes. When these co-transfected cardiomyocytes were irradiated with 980 (Jaws activation) or 808 nm (VChR1 activation) light in the presence of the UCNPs, they showed a marked reduction or speeding up of their beating rate, indicating that these particles could be used for bidirectional control of these cells (Fig. 4k). This effect was even more pronounced in the spheroids, where the entire spheroid contracting together slowed down or sped up when irradiated with 980 or 808 nm light (Fig. 4m), indicating the potential of this system for optical cardiac pacing. The tracking of beating rate was done using the TrackMate plugin (Fiji) developed by Tinevez, et al.^34^.

## Discussion

In summary, conventional Er^3+^ doping in UCNPs is always dominated by identical emission colors under 980 nm or 808 nm excitation. Here we have shown a specially designed UCNPs, wherein the energy migration processes can be regulated such that certain energy transitions are favored at specific excitation wavelengths (980 or 808 nm), resulting in red and green orthogonal emissions from a single lanthanide activator, Er^3+^. These unique UCNPs with tissue-penetrable excitation and emission wavelengths provide a robust toolbox for programmable on-demand optical control of two interactive light-activated channels Jaws and VChR1 in the same cell. The therapeutic potential of these particles for bidirectional control of biological processes is demonstrated here through cardiac pacing. The aforementioned light-gated ion channels when activated by the orthogonal emissions of the UCNPs, control opposing processes, speeding up or slowing down the beating rate of the cardiomyocytes, on demand. We believed that our demonstration herein provides a major step forward towards programmable multi-directional pathway control, and also offers intriguing opportunities for applications in many other synergistically interactive biological processes such as diagnostics and therapies.

## Methods

### Materials

Rare- earth oxides, Y_2_O_3_, Yb_2_O_3_, Er_2_O_3_, Tm_2_O_3_ and Nd_2_O_3_ were purchased from Aladdin (Shanghai, China). These rare-earth oxides were dissolved in acetic acid and kept aside for about 1 h to obtain their acetates. NaF, acetic acid and other commonly used solvents were obtained from Sinopharm Chemical Reagent Co., Ltd. (Shanghai, China). Oleic acid (OA) and octadecene (ODE) were purchased from Sigma–Aldrich. All reagents were used as received without further purification.

### Synthesis of Tm^3+^ doped orthogonal UCNPs

Eight-forty milligram of NaF, 0.8 mmol of Er(CH_3_CO_2_)_3_, 0.195 mmol of Yb(CH_3_CO_2_)_3_, and 0.005 mmol of Tm(CH_3_CO_2_)_3_ were added into 20 mL of OA/ODE(v/v = 1:1) and degassed at 150 °C in vacuum for 0.5 h. The temperature was then rapidly increased to and kept at 300 °C for about 1 h in a N_2_ atmosphere. Meanwhile, 0.9 mmol of Y(CH_3_CO_2_)_3_ and 0.1 mmol of Yb(CH_3_CO_2_)_3_ were added to 8 mL of OA/ODE (v/v = 1:1). This inner shell precursor was degassed and kept at 220 °C in N_2_ atmosphere, and then slowly injected into the core reaction. After maintaining the reaction at 300 °C for 40 min, a pre-prepared outer coating mixture of 0.9 mmol of Nd(CH_3_CO_2_)_3_ and 0.1 mmol of Yb(CH_3_CO_2_)_3_ in 8 mL of OA/ODE was added dropwise into the above reaction, and maintained for another 50 min at 300 °C. The final product solution was mixed with equal volumes of ethanol at room temperature to precipitate the nanoparticles, which were then isolated after centrifugation and washing with a hexane/ethanol mixture.

### Silica coating

The UCNPs were coated with a silica layer as follows: 1 mL of Igepal CO-520 and 18.4 mL of cyclohexane were added to 1.6 mL of 0.05 M UCNPs, homogenized under ultrasonication. To the solution, 160 μL of 30% NH_3_.H_2_O and 40 μL of TEOS (tetraethyl orthosilicate) were added and the mixture shaken for 2 days. The resulting silica-coated UCNPs were purified using acetone and ethanol, and then dispersed in water for further use.

### Cell viability assay

HEK293T cells exposed to different conditions (incubation with varying UCNPs concentrations, exposure to varying 808 and 980 nm irradiation durations etc.) and tested for cell viability after 24 h. Cell viability was assayed using CellTiter 96^®^ AQ_ueous_ One Solution Cell Proliferation Assay (Promega, Madison, WI, USA) as per manufacturer’s instructions.

### Transfection of HEK293T cells with optogenes

fck-Jaws-GFP-ER2 was a gift from Edward Boyden (Addgene plasmid # 65013) and pcDNA3.1/VChR1-EGFP was a gift from Karl Deisseroth (Addgene plasmid # 20955). The plasmids (in *E. coli*) were expanded and purified using Maxiprep (Qiagen plasmid maxi kit, cat. no. 12162). A commercial transfection reagent, Lipofectamine 2000 (Life technologies) was used, for the transfection of HEK293T cells, as per manufacturer’s instructions. Twenty-four hour after transfection, cells were re-plated at low seeding densities (5000 cells per plate of a 24-well plate) and used for further experiments. A commercial transfection reagent, Lipofectamine 3000 (Life technologies) was used for cardiomyocyte transfection,. The concentration of lipofectmine was optimized at 2.5–3 μL lipofectamine3000/ 50 μL optimem. The cells were used for further studies, 48 h post-transfection.

### NIR heating studies

To study the heating effect of 808 and 980 nm NIR laser, different samples such as plain water, cell culture media and cells with culture media were irradiated with both the lasers. For water and cell culture media, 500 μL of each was taken in a 24-well microplate and subjected to irradiation. For the sample with cells, HEK293T cells were grown to confluence on a 24-well microplate with 500 μL of cell culture media and the sample was irradiated for different durations. The increase in temperature was noted using a NIR thermometer. For the cell study, the effect of heat was inferred by measuring cell viability the next day. All the readings were taken in triplicates.

### Cellular uptake of UCNPs

HEK293T cells were grown to confluence in 24-well plates and incubated with silica-coated UCNPs (1 mg/mL) for different durations (10, 60, 120, 240, 480 min) at 37 °C. The cells were then washed twice and the wash solution along with the supernatant was collected for measuring the extracellular concentration of UCNPs. The cells were trypsinized and resuspended in media for measuring the intracellular concentration of UCNPs. The fluorescence emission of the UCNPs was recorded using a Hitachi F-500 fluorescence spectrophotometer (Hitachi High-Technologies Corporation, Tokyo, Japan) equipped with an NIR continuous-wave laser with emission at 980 nm (Photonitech (Asia) Pte. Ltd., Singapore).

### Cellular localization of UCNPs

HEK293T (ATCC^®^ CRL-3216^™^, obtained from American Type Culture Collection, VA, US) cells were grown to confluence in eight-well chamber slides and incubated with 1 mg/mL of UCNPs and incubated at 37 °C for 10–60 min. At the end of incubation, the supernatant was replaced with fresh media and the cells were imaged using a confocal microscope to observe the localization of UCNPs. 2D images and 3D Z-stack images were obtained using the same.

### Measurement of intracellular calcium levels in HEK293T cells

Rhod-4 is a calcium sensitive dye, which fluoresces with a higher intensity when bound to calcium ions. Quest Rhod-4 AM was obtained from AAT Bioquest, Inc. It was dissolved in DMSO to prepare a working solution of 1–10 × 10^−6^ M in Hanks Balanced Salt Solution (HBSS) with 0.02% Pluronic F-127. A working concentration of 5 μM was used for HEK293T cells. Post-transfection with VChR1, the cells were washed and incubated with the dye for 15 min. Following this, the cells were washed and resuspended in media to which UCNPs (1 mg mL^−1^) was added. Through confocal microscopy, Rhod-4 was imaged in the cells with 514 nm excitation and 650 nm emission filters. The cells were then irradiated with NIR laser (808 nm) for 1 min and then imaged again. The change in fluorescence intensities before and after irradiation was analyzed using Matlab and Image-J.

### Measurement of intracellular chloride concentration in HEK293T cells

MQAE (*N*-(ethoxycarbonylmethyl)-6-methoxyquinolinium bromide) is a fluorescent indicator for intracellular chloride ion concentration. The dye detects chloride ions via diffusion-limited collisional quenching, i.e., the fluorescence of the dye quenches according to the intracellular chloride concentration. A 5 mM working solution of MQAE was prepared in HBSS. Post-transfection with Jaws, the cells were washed and incubated with the dye for 30 min. Following this, the cells were washed and resuspended in media to which UCNPs (1 mg mL^−1^) was added. Through confocal microscopy, MQAE was imaged in the cells with 408 nm excitation and 488 nm emission filters. The cells were then irradiated with NIR laser (980 nm) for 1 min and then imaged again. The change in fluorescence intensities before and after irradiation was analyzed using Matlab and Image-J.

### Programmable optogenetics activations experiments

The orthogonal emission UCNPs were first coated with a silica shell. After the fck-Jaws-GFP-ER2 and pcDNA3.1/VChR1-EYFP transfected into HEK293T cells, silica-coated UCNPs were incubated with the cells, and different laser powers and illumination times of 980 nm and 808 nm lasers were used to study the programmable activation of these two synergistic optogenes.

### Electrophysiology study in HEK293T cells

VChR1 and Jaws were transiently transfected into HEK293T cells using Lipofectamine2000 (Life Technologies). Cells were seeded onto Poly-D-Lysine coated coverslips 24 h post-transfection. Whole cells patch-clamp was performed 24 h post seeding (48 h post-transfection). Photocurrents of transient transfected HEK293T were recorded using conventional whole-cell patch-clamp methods. Patch pipettes were pulled from borosilicate glass capillaries and had resistances of 2–3 MΩ when filled with (in mM): K-gluconate, 130; K-Cl, 10.00; EGTA, 5; N-(2-hydroxyethyl) piperazine-N’-ethanesulfonic acid (HEPES); Na3GTP, 0.5; MgATP, 4.0; and Na-Phosphocreatine, 10.0. The external solution contained (in mM): NaCl, 125; KCl, 2.5; Na-Gluconate, 25; CaCl_2_, 1.8; MgCl_2_, 1.0; glucose, 11; HEPES, 10 adjusted with NaOH to pH 7.4 (310 mOsm). Whole-cell currents obtained under voltage clamp with an Multiclamp 700B amplifier (Molecular Device), were filtered at 1–5 kHz and sampled at 5–50 kHz, and the series resistance was typically <5 MΩ.

For the electrophysiology study, the UCNPs were encapsulated inside a PDMS disc which was placed under the coverslip containing the cells. The disc was 1 cm in diameter and about 2 mm thick, containing 5 mg UCNPs. PDMS disc was formed by mixing the monomer, curing agent (Sylgard 184 silicone elastomer kit, Dow Corning) and UCNPs solution (in cyclohexane) in an 8:1:1 ratio, degassing it for 30 mins and then baking it at 70 °C for 1 h.

### Human induced pluripotent stem-cell maintenance and cardiac differentiation

Induced pluripotent stem cells (iPSCs) were derived from a healthy individual as reported previously^35^ and maintained in mTeSR (Stemcell Technologies, Vancouver, Canada) under feeder-free conditions. Human iPSCs were differentiated into cardiomyocytes using a previously described cardiac EB-based protocol^36^. Briefly, the day prior to differentiation, iPSCs were treated with 10 µM ROCK inhibitor Y-27632 (Calbiochem, CA, USA). The following day, iPSCs were dissociated into single cells using Accutase (Stemcell Technologies) and resuspended in mTeSR:DMEM/F12-B27 (1:1) medium supplemented with PVA (4 mg/ml), ascorbic acid (284 µM) and BMP-4 (770pM) to form 5000 cell EBs in AggreWells (Stemcell Technologies). The following day, aggregated EBs were removed from the AggreWells and maintained (in suspension) in mTeSR:DMEM/F12-B27 (1:4) + PVA medium supplemented with ascorbic acid (284 µM) and BMP-4 (1.5 nM) and additional activin A (1.5 nM), FGF2 (3.1 nM) and SB203580 (5 µM) for 72 h, after which they were maintained in DMEM/F12-B27 + PVA medium supplemented with ascorbic acid (284 µM), SB203580 (5 µM), VEGF (1.5 nM), cyclosporine A (2.5 µM), IWP-4 (10 µM), noggin (4.3 nM), and A83-01 (1 µM) for 48 h. Finally, the EBs were maintained in the above DMEM/F12-B27 + PVA medium supplemented with ascorbic acid (284 µM), SB203580 (5 µM), VEGF (521pM), cyclosporine A (2.5 µM), and IWP-4 (10 µM) for 48 h, after which they were plated on 0.1% gelatin-coated plates and maintained as cardiac spheroids in DMEM (Thermo Fisher Scientific, MA, USA) supplemented with 2% FBS, non-essential amino acids and GlutaMAX (Thermo Fisher Scientific). For experiments involving single cells, cardiac spheroids were dissociated using Embryoid Body Dissociation Kit (Miltenyi Biotec, Bergisch Gladbach, Germany) and plated on 0.1% gelatin-coated plates. Growth factors were precured from R&D Systems (MN, USA), small molecules from Calbiochem and chemicals from Sigma–Aldrich (MO, USA).

### Bidirectional control of cardiomyocytes

Induced pluoripotent stem-cell (iPSCs) derived cardiomyocytes were prepared as beating clusters of 200–500 μm size. Cardiomyocytes were also obtained from STEMCELL technologies. Cells from these sources were used for the spheroid and single cell studies, respectively. Transfection with Jaws and VChR1 plasmids was done using Lipofectime 3000. Cells were plated on 35 mm dishes coated with 1% gelatin and maintained using DMEM supplemented with 2% fetal bovine serum, non-essential amino acids and glutamax (Life technologies). UCNPs were encapsulated inside a PDMS disc which was placed under the dish containing the cells. The disc was 1 cm in diameter and about 2 mm thick, containing 15 mg UCNPs. PDMS disc was formed by mixing the monomer, curing agent (Sylgard 184 silicone elastomer kit, Dow Corning) and UCNPs solution (in cyclohexane) in an 8:1:1 ratio, degassing it for 30 mins and then baking it at 70 °C for 1 h. The beating rate of the cells was tracked using the TrackMate plugin in Fiji, developed by Tinevez et al.^1^.

### Characterization of UCNPs

Transmission electron microscopy (TEM) images were recorded on a JEOL 2010F transmission electron microscope (Jeol Ltd., Tokyo, Japan) operating at an acceleration voltage of 200 kV. Fluorescence spectra of UCNPs were recorded on a PE LS-55 fluorescence spectrophotometer equipped with an NIR continuous-wave laser with emission at 980 nm or 808 nm.

### Statistical analysis

To compare the mean values of experimental group to that of control ones, one-way ANOVA, at an alpha level of 0.01 (a *P*-value of less than 0.01 is considered as statistically significant), was performed using OriginPro 8.5.

### Reporting summary

Further information on research design is available in the Nature Research Reporting Summary linked to this article.

## Supplementary information

Supplementary Information

Reporting Summary

## Data Availability

The data sets generated during and/or analyzed during the current study are available from the corresponding author on reasonable request.
