# Supplementary material for: Manipulating energy migration within single lanthanide activator for switchable upconversion emissions towards bidirectional photoactivation
Source: Nat Commun. 2019 Sep 27;10:4416. doi: 10.1038/s41467-019-12374-4 (PMC6764961; doi:10.1038/s41467-019-12374-4)
Supplement: Supplementary file 1 — Supplementary Information [file 41467_2019_12374_MOESM1_ESM.pdf]

Supplementary Information

Manipulating Energy Migration within Single Lanthanide Activator for Switchable Upconversion Emissions Towards Bidirectional Photoactivation

Qingsong Mei,[1] Akshaya Bansal,[1] Muthu Kumara Gnanasammandhan Jayakumar,[1] Zhiming Zhang,[2] Jing Zhang,[2] Hua Huang,[3,4] Dejie Yu,[3] Chrishan J.A. Ramachandra[5,6], Derek J. Hausenloy [5-10], Tuck Wah Soong,[3,11] Yong Zhang[1]*

[1] *Department of Biomedical Engineering, Faculty of Engineering, National University of Singapore, Singapore 117583, Singapore*

[2] *School of Environmental and Chemical Engineering, Shanghai University, 99 Shangda Road, 200444, Shanghai, China*

[3] *Department of Physiology, Yong Loo Lin School of Medicine, National University of Singapore, Singapore 117456, Singapore*

[4] *Electrophysiology core, Medical Science cluster, Yong Loo Lin School of Medicine, National University of Singapore, Singapore 117456, Singapore*

[5] *Cardiovascular & Metabolic Disorders Program, Duke-National University of Singapore Medical School, Singapore*

[6] *National Heart Research Institute Singapore, National Heart Centre, Singapore*

[7] *Yong Loo Lin School of Medicine, National University Singapore, Singapore*

[8] *The Hatter Cardiovascular Institute, University College London, London, UK*

[9] *The National Institute of Health Research University College London Hospitals Biomedical Research Centre, Research & Development, London, UK*

[10] *Tecnologico de Monterrey, Centro de Biotecnologia-FEMSA, Nuevo Leon, Mexico*

[11] *Neurobiology/Ageing Programme, Life Sciences Institute, National University of Singapore, Singapore 117456, Singapore*

* Corresponding author. Email: biezy@nus.edu.sg (Y.Z.)

1. **Supplemental data for Tm^{3+} doped UCNPs.**

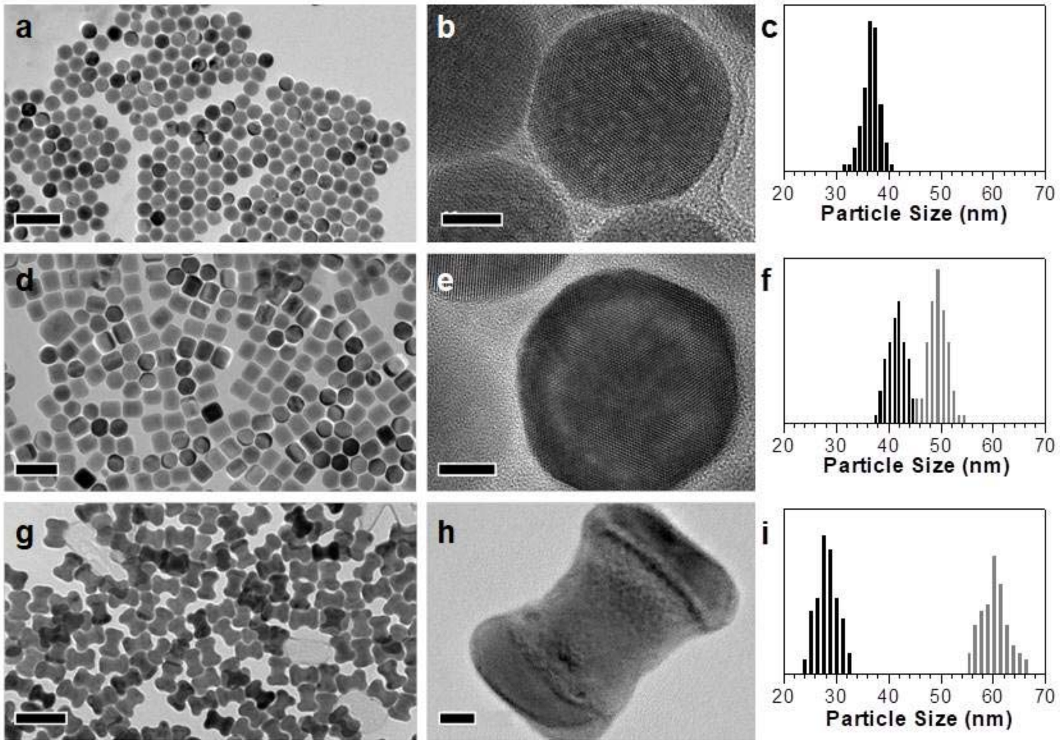

Supplementary Figure 1. TEM images and size distributions of the core NPs $NaErF_4$:Yb/Tm (a-c), core-shell NPs $NaErF_4$:Yb/Tm @ $NaYF_4$:Yb (d-f), and the core-shell-shell NPs $NaErF_4$:Yb/Tm @ $NaYF_4$:Yb @ $NaNdF_4$:Yb (g-i). Scale bars in a, d and g are 100 nm. Scale bars in b, e and h are 10 nm.

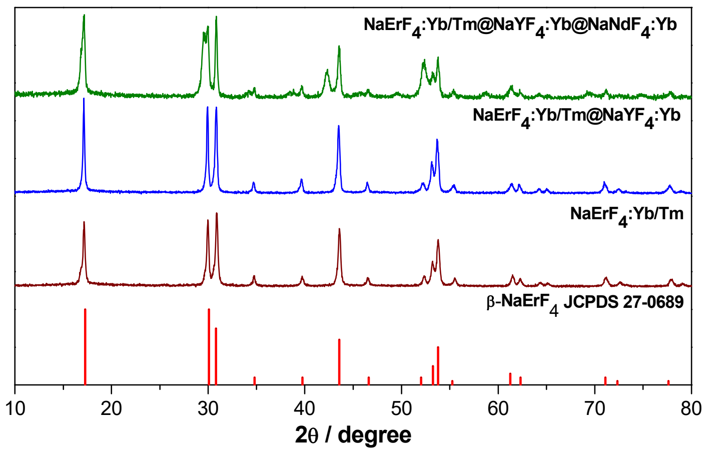

Supplementary Figure 2. XRD patterns of the the core NPs $NaErF_4$:Yb/Tm, core-shell NPs $NaErF_4$:Yb/Tm @ $NaYF_4$:Yb, and the core-shell-shell NPs $NaErF_4$:Yb/Tm @ $NaYF_4$:Yb @ $NaNdF_4$:Yb.

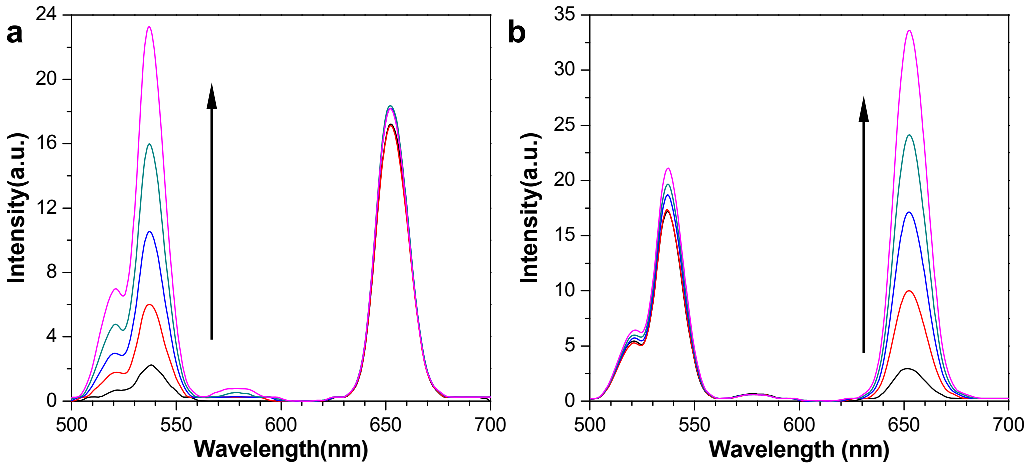

Supplementary Figure 3. (a) Luminescence spectra of the UCNPs $NaErF_4$:Yb/Tm @ $NaYF_4$:Yb @ $NaNdF_4$:Yb in cyclohexane when excited at a fixed 980 nm laser power (1 W), and the 808 nm laser power increased from 0 W to 1.5, 2, 2.5 and 3 W from bottom to top. (b) Luminescence spectra of the UCNPs when excited at a fixed 808 nm laser power (2 W), and the 980 nm laser power changed from 0 W to 1, 1.5, 2 and 2.5 W from bottom to top.

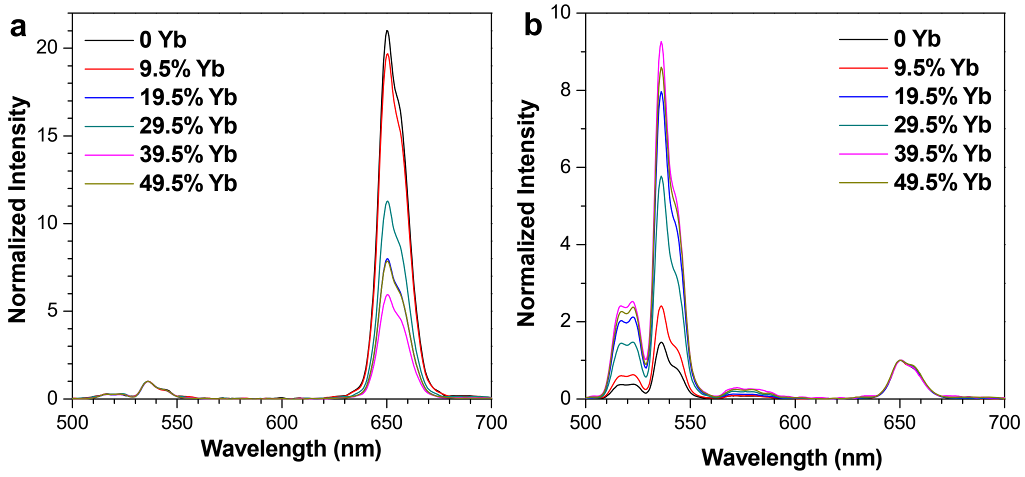

Supplementary Figure 4. Normalized upconversion luminescence spectra of the UCNPs $NaErF_4:Yb^{3+}$ (x%)/Tm^{3+} (0.5%) @ $NaYF_4:Yb^{3+}$ (10%) @ $NaNdF_4:Yb^{3+}$ (10%) with different Yb^{3+} doping amounts in the core under 980 nm (a) and 808 nm (b) lasers excitation.

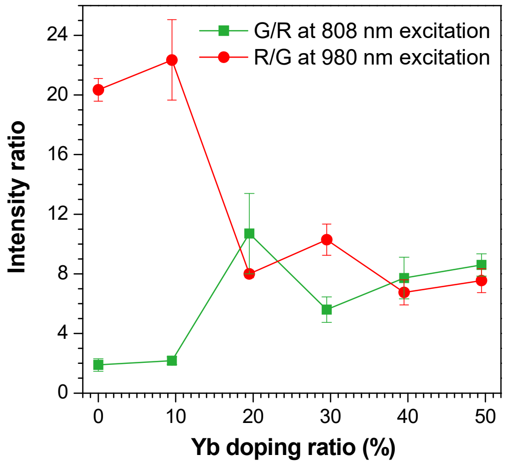

Supplementary Figure 5. Intensity ratio of red to green under 980 nm (red line), and green to red under 808 nm excitation (green line) of the UCNPs $NaErF_4:Yb^{3+}$ (x%)/Tm^{3+} (0.5%) @ $NaYF_4:Yb^{3+}$ (10%) @ $NaNdF_4:Yb^{3+}$ (10%) with different Yb^{3+} doping amounts. Error bars represent the standard deviation of ratio measurements of three batches of UCNPs.

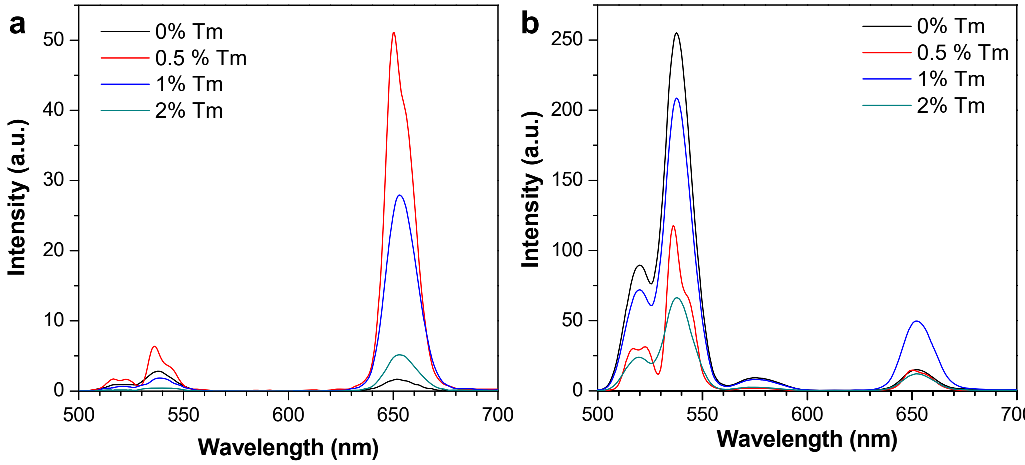

Supplementary Figure 6. Upconversion luminescence spectra of the UCNPs $NaErF_4:Yb^{3+}$ (19.5%)/Tm^{3+} (x%) @ $NaYF_4:Yb^{3+}$ (10%) @ $NaNdF_4:Yb^{3+}$ (10%) with different Tm^{3+} doping amounts in the core under 980 nm (a) and 808 nm (b) lasers excitation.

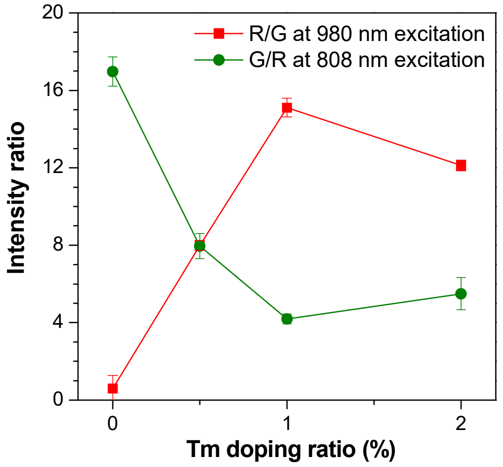

Supplementary Figure 7. Intensity ratio of red to green under 980 nm (red line), and green to red under 808 nm excitation (green line) of the UCNPs $NaErF_4:Yb^{3+}$ (19.5%)/Tm^{3+} (x%) @ $NaYF_4:Yb^{3+}$ (10%) @ $NaNdF_4:Yb^{3+}$ (10%) with different Tm^{3+} doping amounts. Error bars represent the standard deviation of ratio measurements of three batches of UCNPs.

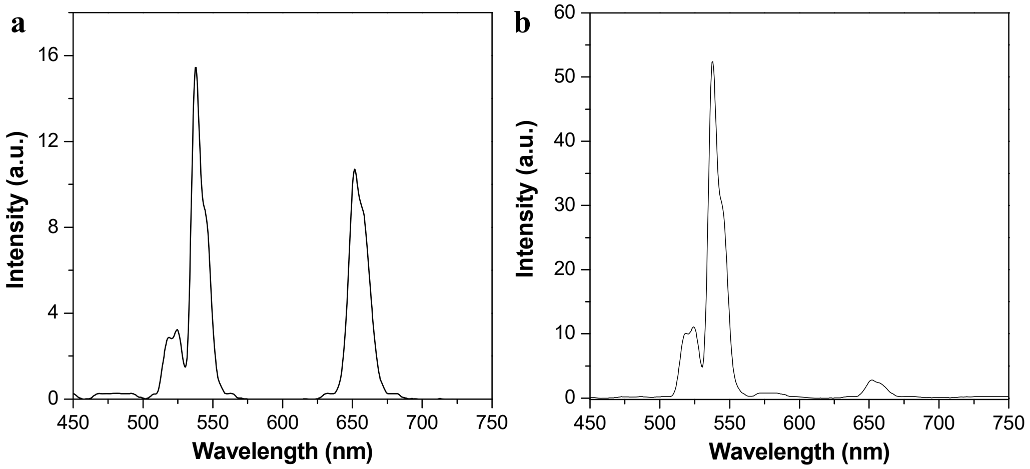

Supplementary Figure 8. Upconversion luminescence spectra of the UCNPs $NaErF_4:Yb^{3+}$ (19.5%) @ $NaYF_4:Yb^{3+}$ (10%) @ $NaNdF_4:Yb^{3+}$ (10%) under 980 nm (a) and 808 nm (b) lasers excitation.

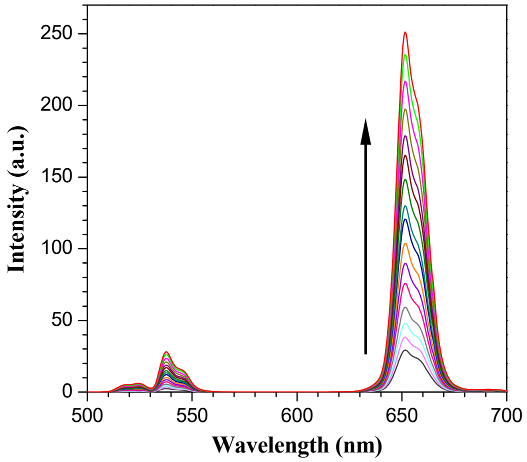

Supplementary Figure 9. Dependence of luminescence intensities of the UCNPs $NaErF_4$:Yb^{3+}/Tm^{3+} (19.5/0.5%) @ $NaYF_4$:Yb^{3+} (10%) @ $NaNdF_4$:Yb^{3+} (10%) on 980 nm excitation laser power from 1 W to 4 W.

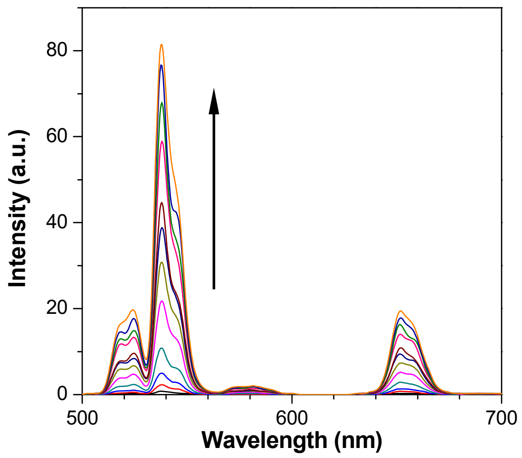

Supplementary Figure 10. Dependence of luminescence intensities of the UCNPs $NaErF_4$:Yb^{3+}/Tm^{3+} (19.5/0.5%) @ $NaYF_4$:Yb^{3+} (10%) @ $NaNdF_4$:Yb^{3+} (10%) on 808 nm excitation laser power from 1 W to 6 W.

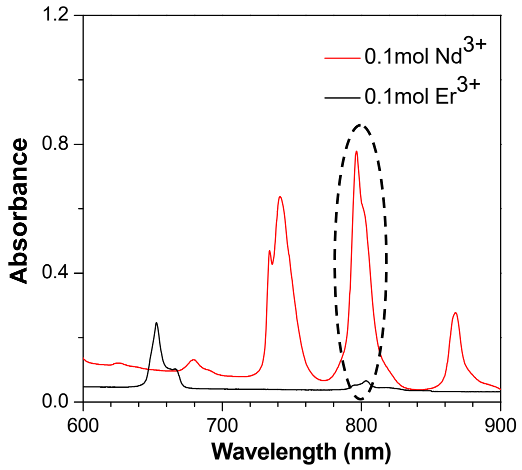

Supplementary Figure 11. Absorption spectra of equal amounts of Er^{3+} aqueous solution (black line), and Nd^{3+} aqueous solution (red line).

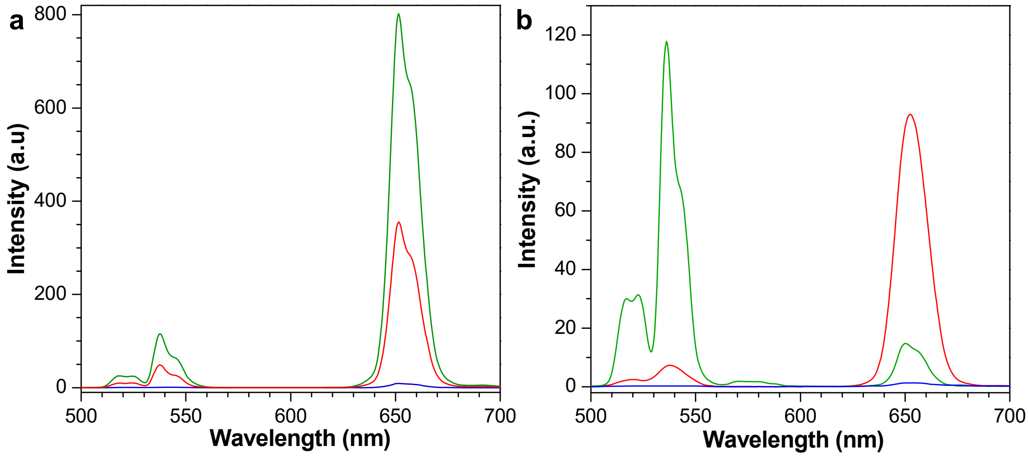

Supplementary Figure 12. Upconversion luminescence spectra of the core NPs NaErF4:Yb^{3+}/Tm^{3+} (19.5/0.5%) (blue line), core-shell NPs NaErF4:Yb^{3+}/Tm^{3+} (19.5/0.5%) @ NaYF4:Yb^{3+} (10%) (red line), and core-shell-shell NPs NaErF4:Yb^{3+}/Tm^{3+} (19.5/0.5%) @ NaYF4:Yb^{3+} (10%) @ NaNdF4:Yb^{3+} (10%) (green line) under 980 nm excitation (a), and 808 nm excitation (b).

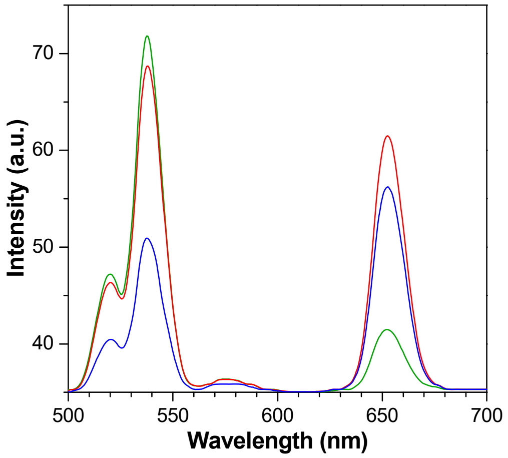

Supplementary Figure 13. Luminescence spectra of the core-shell-shell structured UCNPs $NaErF_4:Yb^{3+}/Tm^{3+}$ (19.5/0.5%) @ $NaYF_4:Yb^{3+}$ (x%) @ $NaNdF_4:Yb^{3+}$ (10%) with different Yb^{3+-} doping amounts in the first shell under 808 nm excitations. The green, red and blue lines are 10%, 20%, and 30%, respectively.

Supplementary Table 1. Absolute upconversion quantum yields of the as-prepared Tm doped UCNPs.

	Quantum Yield (%)	Excitation Wavelength (nm)	Excitation Power (W)
Tm doped	0.086	980	0.5
UCNPs	0.074	808	1.5

These measured quantum yield values are comparable to previously reported values of UCNPs exited with 980 nm or 808 nm irradiations.[2]

2. Supplemental data for photoactivation experiments.

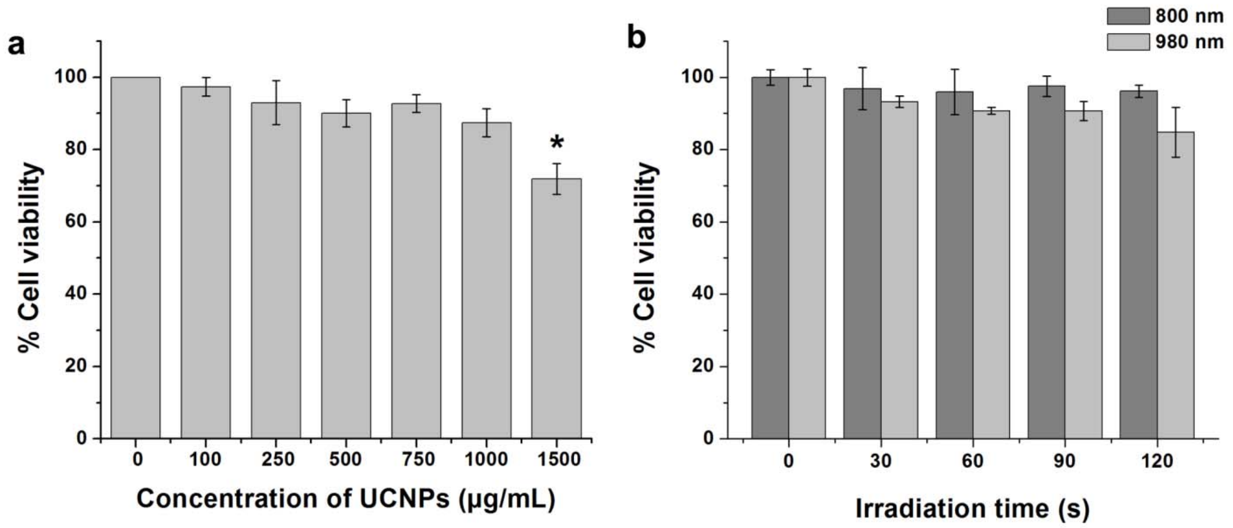

Supplementary Figure 14. (a) Viability of HEK293T cells treated with different concentrations of silica coated UCNPs. (b) Viability of the cells exposed to different durations of 980/808 nm pulsed (100ms pulse width, 1 Hz) NIR laser. Error bars represent the standard deviation of measurements , *p<0.01(ANOVA)

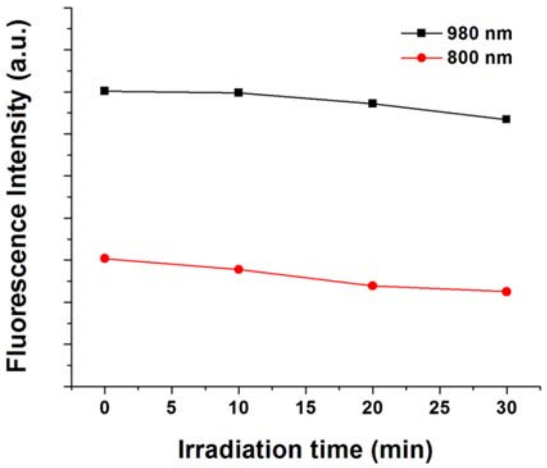

Supplementary Figure 15. Photostability of UCNPs on continuous irradiation with 980 and 808 nm (100ms pulse width, 1 Hz).

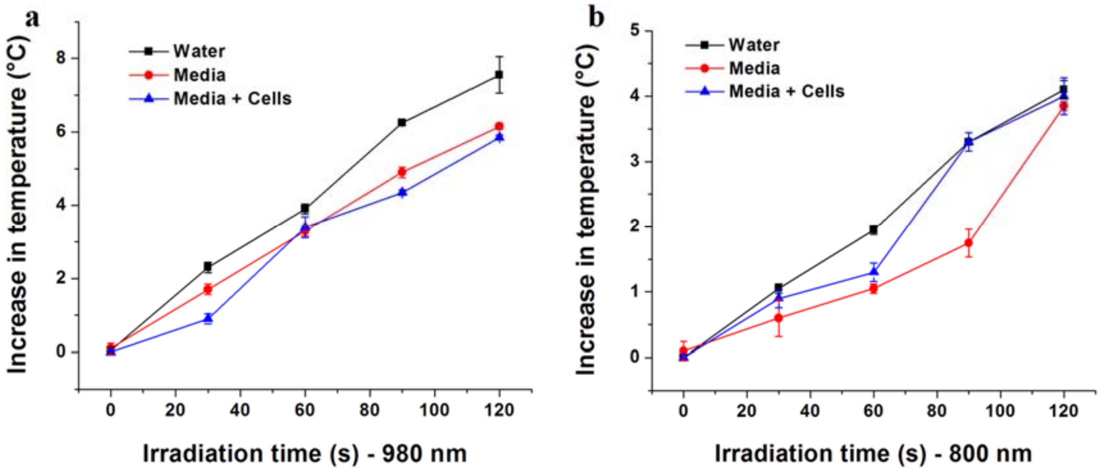

Supplementary Figure 16. Increase in temperature of different substrates when exposed to 980 nm (a) and 808 nm (b) NIR laser irradiation. Error bars represent the standard deviation of measurements

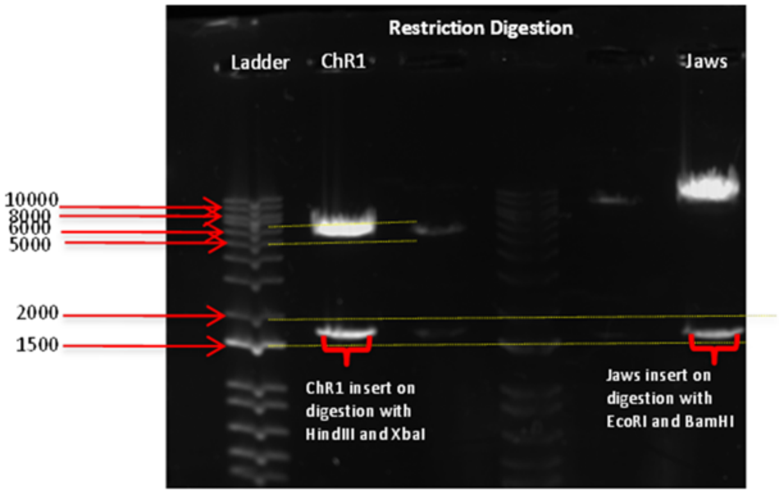

Supplementary Figure 17. Gel showing bands formed after restriction digestion of Jaws and ChR1 plasmids with enzymes targeted to their respective insert flanking restriction sites. The expected insert size for ChR1 and Jaws is 1550 and 1630 base pairs respectively. This is what we see in the gel. This is confirmation that these plasmids do indeed contain the requisite inserts for expressing the ChR1 and Jaws channels. This combined with GFP imaging of cells post transfection, confirm that the plasmids are intact and functional.

Following plasmid expansion and purification, restriction digestion was done to ensure that the inserts coding for the opsins were present in the plasmids isolated. In the fck-Jaws-GFP-ER2 plasmid, the Jaws-GFP-ER2 coding fragment (1584 bp) was inserted between the BamHI and EcoRI cloning sites. In the pcDNA3.1/VChR1-EYFP plasmid, an insert of size 1626 bp was cloned between the HindIII and XbaI cloning sites, For restriction digestion, the corresponding restriction enzymes were used (BamHI, EcoRI for Jaws plasmid and HindIII and XbaI for the VChR1 containing plasmid). Figure S26 shows the gel obtained upon loading the DNA products post digestion. The 1% agarose gel shows the expected number of bands for both plasmids (2 for both) with the expected sizes. Both the plasmids show inserts in the 1500-2000 bp size range, confirming that the opsin coding inserts are present in both plasmids. Figure S27 shows expression of EYFP and GFP in HEK293T cells transfected with the pcDNA3.1/VChR1-EYFP and fck-Jaws-GFP-ER2 plasmids, respectively. The expression of these fluorescent tags also confirms that the isolated plasmids are intact and functional, since these markers are fused to the opsins and are expressed together.

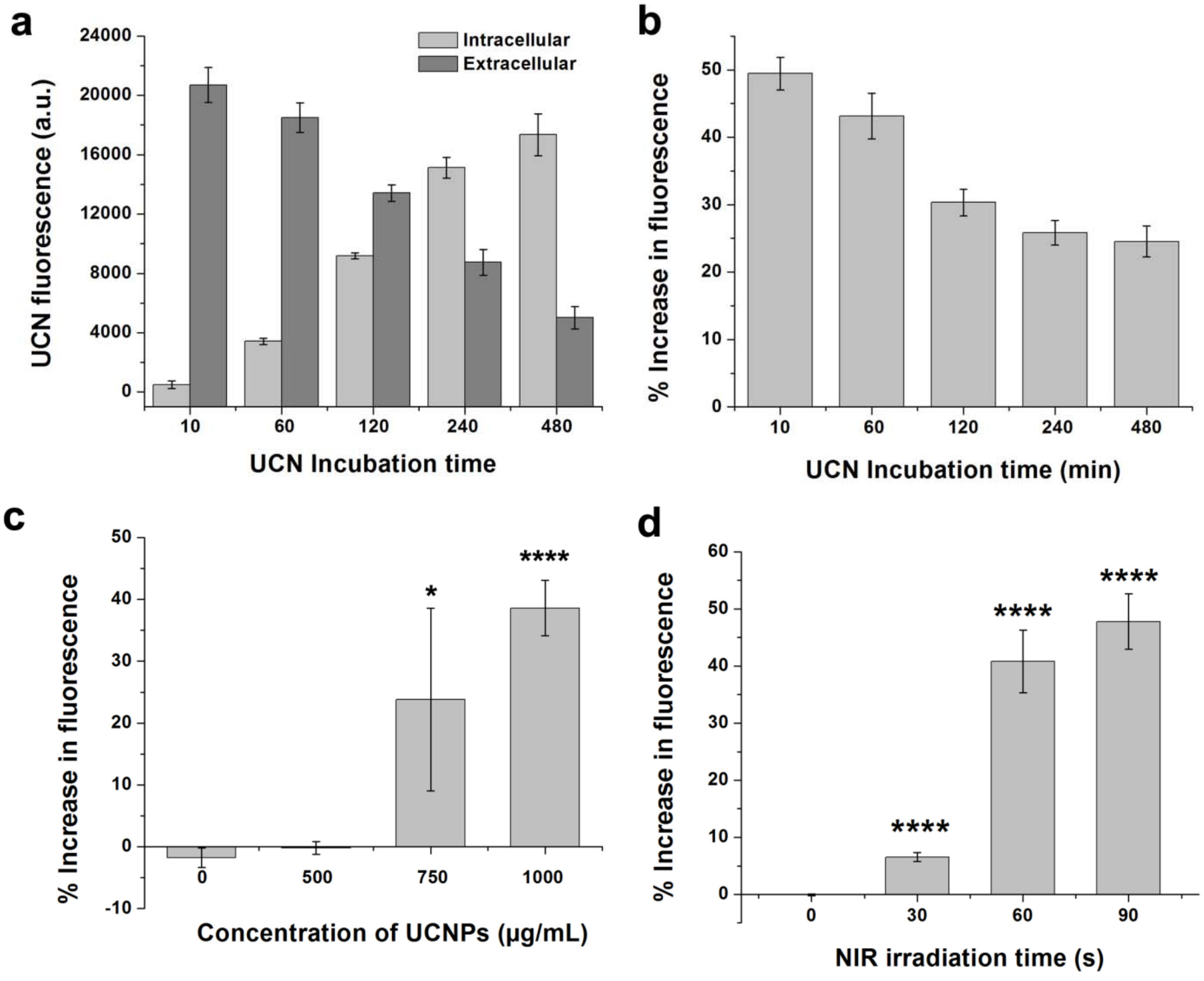

Supplementary Figure 18. (a) Intracellular and extracellular luminescence intensities of UCNPs incubated with HEK239T cells over 8 hours. (b) Change in Rhod-4 fluorescence in HEK293T cells incubated with UCNPs for varying durations of time and irradiated with NIR light at 980 nm. Increase in Rhod-4 fluorescence is an indicator of VChR1 activation. (c) Changes of Rhod-4 fluorescence in VChR1 transfected cells with varying UCNPs concentrations, and (d) durations of NIR light exposure. Error bars represent the standard deviation of measurements, *p<0.01 (ANOVA), ****p<0.00001 (ANOVA).

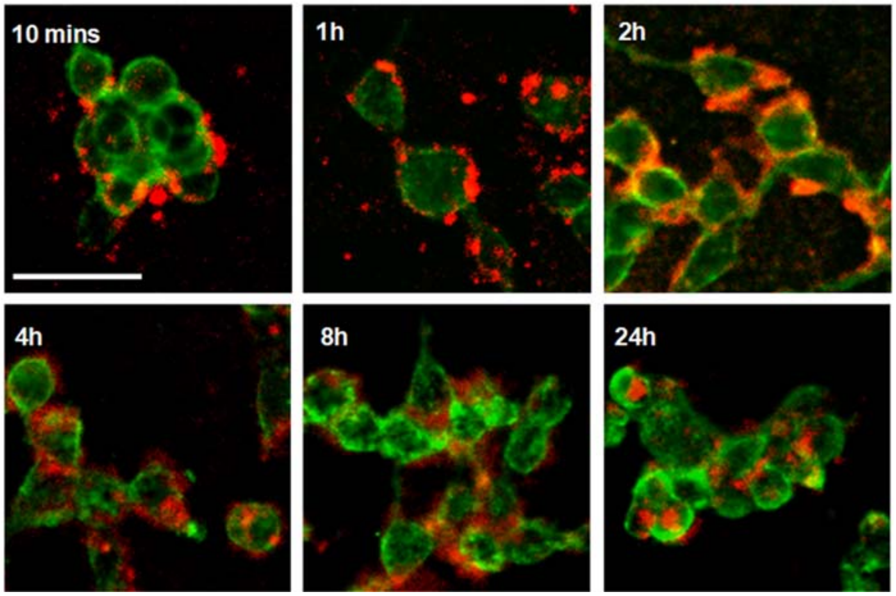

Supplementary Figure 19. Merged fluorescence images taken using a confocal microscope showing uptake and distribution of UCNPs in HEK293T cells at incubation durations ranging from 10 mins to 24 hours. The cell membrane is stained using Concanavalin A (green) and the UCNPs are shown in red. Scale bar is 50 µm.

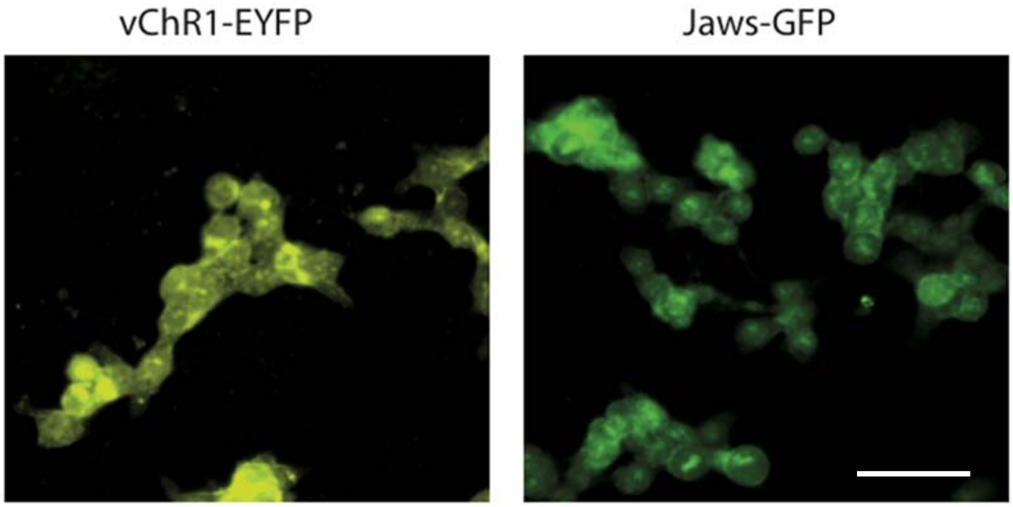

Supplementary Figure 20. Transfection and expression of VChR1 and Jaws in HEK293T cells as seen by the co-expression of GFP and YFP respectively. Scale bar is 50 µm.

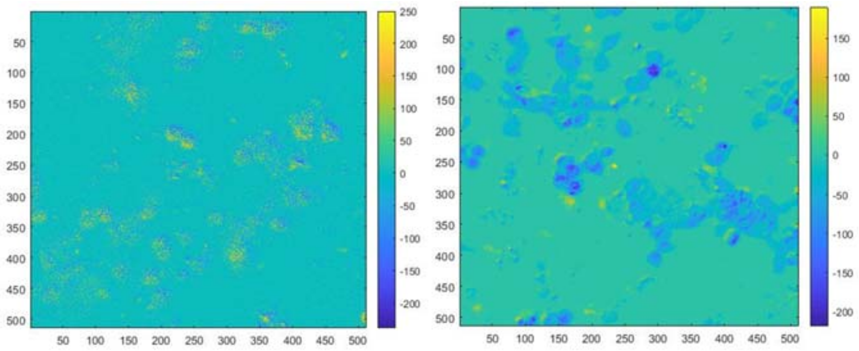

Supplementary Figure 21. Representative subtraction images showing changes in Rhod-4 and MQAE fluorescence intensity before and after irradiation with 980 nm and 808 nm CW lasers in cells transfected with VChR1 (left) and Jaws (right), respectively. x and y-axes show image scales in µm.

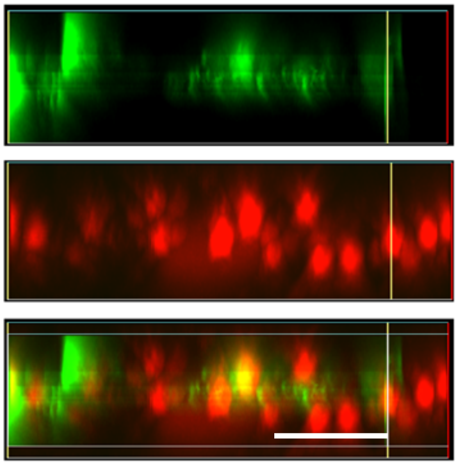

Supplementary Figure 22. Confocal Z-stack images showing distribution of UCNPs in and around cells after 60 mins of incubation. The top image shows Con-A staining (cell membrane), the middle image shows UCNPs fluorescence and the bottom image is merged composite of the two, showing surface localisation. Scale bar is 50 μm.

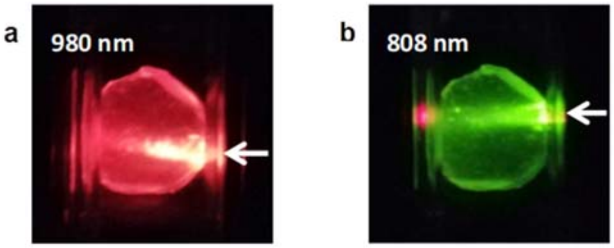

Supplementary Figure 23. Luminescent images of a PDMS disc containing orthogonal emission UCNPs under excitation with 980 nm laser (a) and 808 nm laser (b).

Supplementary references:

1. Tinevez, J.-Y. *et al.* TrackMate: An open and extensible platform for single-particle tracking. *Methods* **115**, 80-90 (2017).
2. Deng, R. R. *et al.* Temporal full-colour tuning through non-steady-state upconversion. *Nature Nanotechnology* **10**, 237-242 (2015).
